# Immune checkpoint inhibitors in cancer patients with COVID-19

**DOI:** 10.1515/biol-2022-0641

**Published:** 2023-07-07

**Authors:** Yun Pan, Jiaxiong Tan, Jinzhong Li, Taoyuan Li, Jieying Li, Yang Cao, Liu Yang, Xunge Lin, Minran Li, Xujing Liang

**Affiliations:** Department of Infectious Disease, First Affiliated Hospital, Jinan University, Guangzhou, 510632, Guangdong, China; Institute of Hematology, School of Medicine, Key Laboratory for Regenerative Medicine of Ministry of Education, Jinan University, Guangzhou, 510632, Guangdong, China

**Keywords:** immune checkpoint inhibitors, immunotherapy, COVID-19

## Abstract

Immune checkpoint inhibitors (ICIs) are widely used to treat a variety of cancers and common infectious diseases with high efficacy. During the coronavirus disease 2019 (COVID-19) pandemic, studies suggested that COVID-19 patients may benefit from ICI immunotherapy. However, clinical studies on the safety and efficacy of ICI in COVID-19 patients are still being conducted. Currently, it is not clear whether cancer patients undergoing ICI immunotherapy should adjust their treatment strategy after infection with SARS-CoV-2 and whether ICI can reduce the viral load of severe acute respiratory syndrome coronavirus 2 (SARS-CoV-2). In this study, reports of patients with different types of tumors infected with SARS-CoV-2 under ICI immunotherapy were classified and sorted, including lung cancer, melanoma, squamous cell carcinoma of the head and neck, and hematologic malignances. The safety and efficacy of ICI in antitumor and anti-SARS-CoV-2 therapies were compared and further discussed to provide more reference materials for the application of ICI treatment. In a word, COVID-19 has changed the ICI treatment strategy for cancer patients indeed, and ICI treatment may be a “double-edged sword” for cancer patients complicated with COVID-19.

## 1 Introduction

Immune checkpoints (ICs), such as programmed cell death-1 (PD-1), cytotoxic T-lymphocyte antigen-4 (CTLA-4), T-cell immunoglobulin-3, are expressed in a variety of immune cells, such as T cells, natural killer cells, and dendritic cells, which can trigger immunosuppressive signaling pathways. Under physiological conditions, these molecules and co-stimulatory molecules are strictly regulated to achieve a balance to prevent overactivation of the autoimmune system, leading to tissue damage. In tumors and chronic infectious diseases, upregulated expression of multiple ICs and their ligands may lead to “T-cell exhaustion,” which mediates antigenic immune escape. Based on the immune escape mechanism, ICI immunotherapy has been widely used in several cancer patients and common infectious diseases and has achieved good efficacy [1,2].

ICI immunotherapy has also been widely studied and applied in infectious diseases. In a phase II clinical trial in patients with advanced liver cancer due to hepatitis C cirrhosis, Sangro et al. [3] found that patients achieved better antitumor effects and reduced hepatitis C virus viral load by ICI immunotherapy, without serious adverse events (AEs). Similarly, Cook et al. also showed that ICIs may be a safe and effective treatment in human immunodeficiency virus-infected patients with advanced-stage cancer [4]. In addition to chronic viral infections, high levels of IC molecules have also been observed in the early stages of acute viral infections [5]. Thus, we consider that the most fundamental reason why patients with chronic viral infection and tumor may benefit from IC-based immunotherapy is that numerous ICs pathways *in vivo* mediate immune escape mechanisms, for both virus and tumor antigens. Coronavirus disease 2019 (COVID-19) is an acute infectious disease mainly involving the respiratory system caused by severe acute respiratory syndrome coronavirus 2 (SARS-CoV-2), which has caused a global pandemic and seriously affected normal human life. Studies have pointed out that there is also “T-cell exhaustion,” a serious phenomenon in patients with severe COVID-19, manifested by decreased cytokine secretion ability, low proliferation ability, and accelerated apoptosis of T lymphocytes, accompanied by upregulated expression of ICs and their ligands [6]. Mild and convalescent patients showed the normal function of major immune cells and low IC expression. A series of studies suggest that COVID-19 patients may benefit from ICI [7]. In addition, we should also consider that an overactivated immune system is one of the crucial factors leading to COVID-19 pneumonia. Therefore, whether ICI immunotherapy will mediate the “double hit” of lung inflammation is still not conclusive, but it is worth further discussion. Clinical studies on the safety and efficacy of ICI in COVID-19 patients are still being conducted (e.g., NCT04356508, NCT04335305, NCT04413838, NCT04343144). There are still no public results of clinical studies on antiviral and antitumor effects. Does ICI immunotherapy help reduce the viral load of SARS-CoV-2? Do patients with tumors undergoing ICI immunotherapy need to adjust their treatment strategy after infection with SARS-CoV-2? There are still no positive answers.

Therefore, this study classifies the reports of patients with different types of tumors infected with SARS-CoV-2 receiving ICI treatment, compares and discusses the safety and efficacy of ICI immunotherapy against tumors and SARS-CoV-2, and provides more references for the application of ICI treatment (Table 1).

**Table 1:** Application of ICI in tumor patients with COVID-19

Tumor types	Location	Number of patients	Sex	Age (year)	Immune checkpoint inhibitors	Target (PD-1/PD-l1/CTLA-4)	Antitumor effect (active/negative)	Pneumonia assessment (active/negative)	Author^x^
NSCLC	Germany	1	M	40	Pembrolizumab	PD-1	Active	Negative	Mellinghoff SC [7]
Lung adenocarcinoma	Japan	1	M	70	Nivolumab ipilimumab	PD-1, CTLA-4	Active	Negative	Murata D [18]
Melanoma	Germany	1	F	83	Nivolumab ipilimumab	PD-1, CTLA-4	Active	Negative	Ahmed MS [26]
Melanoma	Germany	1	F	47	Nivolumab	PD-1	Active	Active	Schmidle P [27]
Melanoma	Turkey	1	F	75	Nivolumab	PD-1	Active	Active	Yekedüz E [28]
Melanoma	Italy	1	M	54	Pembrolizumab	PD-1	Active	Active	Pala L [29]
Melanoma	Brazil	1	M	83	Nivolumab ipilimumab	PD-1, CTLA-4	—	Negative	Souza IL [32]
	Brazil	1	F	74			—	Negative	
Melanoma	Germany	5	—	—	Pembrolizumab	PD-1	—	Active	Moritz RKC [24]
		2	—	—	Nivolumab	PD-1	—	Active	
		4	—	—	Nivolumab ipilimumab	PD-1, CTLA-4	—	Active	
		2	—	—	Nivolumab ipilimumab	PD-1, CTLA-4	—	Negative	
Squamous head and neck carcinoma	Italy	1	F	65	—	PD-L1	Active	Negative	Dipasquale A [34]
Hodgkin lymphoma	Ireland	1	F	22	Pembrolizumab	PD-1	Active	Active	O’Kelly B [43]

F: female; M: male.

## 2 Lung cancer with SARS-CoV-2 infection

ICI immunotherapy has changed the treatment pattern of patients with lung cancer and significantly improved prognosis. Nevertheless, studies have shown that age and ICI treatment are related to the severity of COVID-19 [8,9]. In contrast, some studies have pointed out that ICI treatment is not a risk factor for serious development in patients with cancer due to SARS-CoV-2 infection [10,11,12]. A prospective regression analysis of 800 cancer patients by Lee et al. receiving a variety of treatments, including 44 immunotherapies, warrants further attention, which clearly showed no significant difference in mortality between those who received immunotherapy and those who did not (odds ratio: 0.59 [95% CI: 0·27–1·27]; *p* = 0.177) [10]. Luo et al. [13] followed 69 patients with lung cancer infected with COVID-19, including 41 patients who had previously received PD-1 blockade and 28 patients who had not received yet, and found no difference in COVID-19 severity (hospitalization, intensive care unit (ICU)/intubation/transition to do not intubation, and mortality) between these two groups. It demonstrated that PD-1 blockers did not increase COVID-19 severity in patients with lung cancer. In addition, a multicenter observational study showed that systematic antitumor therapy (such as pembrolizumab, nivolumab, and ipilimumab), ICI combination chemotherapy, TKIs, and chemotherapy alone did not affect the survival rate of patients with lung cancer complicated with COVID-19 [14].

Clinicians are discussing the need to adjust the dose and timing of immunotherapy in COVID-19 patients. Sehgal et al. [15] reported no difference in overall survival (OS) or progression-free survival (PFS) in patients with advanced non-small-cell lung cancer (NSCLC) with prolonged pembrolizumab administration intervals compared to standard treatment intervals. Furthermore, Joshi et al. [16] conducted a retrospective analysis of adjuvant durvalumab administration in patients with NSCLC during the COVID-19 pandemic and showed a lower grade 3–4 AEs rate when administered every 4 weeks than when administered every 2 weeks (7 vs. 15%). Similar results were observed by Hijmering-Kappelle, who recommended the continuation of extended ICI drug administration after the COVID-19 pandemic, considering the economic benefits [17]. However, due to the limitations of retrospective analysis, more prospective studies with larger sample sizes are needed further to evaluate the effect of prolonged dosing time on efficacy.

Mellinghoff [7] reports a middle-aged male with NSCLC with multiple distant metastases (brain, liver, bone, pleura, spleen, etc.) who developed severe respiratory symptoms due to cancer progression during COVID-19 infection using pembrolizumab combined with chemotherapy. The disease could not be identified as a progression of COVID-19 or immune-related pneumonitis. After 1 week of empirical hormone therapy, pneumonia gradually subsided, and the tumor became smaller. Interestingly, there was no sign of pneumonia when pembrolizumab was re-initiated, as the overall situation improved. The patient achieved partial remission (PR) at 6 months’ follow-up and could move around independently. The aforementioned reports alert us to additive lung injury attributable to PD-1 monoclonal antibody combined chemotherapy in patients with such tumors.

Another 70-year-old lung adenocarcinoma patient diagnosed with COVID-19 was treated with a combination of two immunotherapeutic agents (nivolumab and ipilimumab) but eventually died from cytokine release syndrome, suggesting that a higher mortality risk may be associated with the combination immunotherapy [18]. However, the case mentioned is an elderly patient, whose final outcome may be complicated by age, organ function, and complications. In addition, as to whether combined immunotherapy increases the mortality rate, the following two related studies may also give similar conclusions [19,20]. In the study by Owonikoko [19], the mortality rate associated with combined therapy is 2.5%, which is significantly higher than that of single drug therapy (0.4%). Immune-related AEs (irAEs) reflect the good curative effect of ICI treatment, but it should also be vigilant that it may endanger life at any time [21]. It is controversial whether ICI therapy should be combined to treat patients with COVID-19. Currently, PD-1 inhibitors combined with CTLA-4 inhibitors are not considered to improve OS and PFS in patients with lung cancer but are associated with greater toxic side effects [19,20].

On the other hand, higher levels of SARS-CoV-2-responsive IgG, neutralizing serum activity, and a stronger humoral immune response were found in a patient with NSCLC who received pembrolizumab in combination with chemotherapy than in a normal COVID-19 patient [7]. Continuous increases in follicular helper T cell and activated CD4+ and CD8+ T cells were also observed, suggesting that humoral immunity and cellular immunity were enhanced.

## 3 Melanoma with SARS-CoV-2 infection

CTLA-4 was first used in melanoma patients and improved prognosis; the subsequent approval of PD-1 inhibitors further extended survival. A Japanese study on cancer statistics indicated that the efficacy of ICIs varied by subtype of melanoma, with a poor response rate for acral and mucosal melanoma and a high response rate for primary hyperplasia of connective tissue melanoma [22]. A retrospective analysis of 50 patients with advanced melanoma with COVID-19 in Spain showed that the mortality rates of patients receiving immunotherapy, targeted therapy, and no treatment were 16, 15, and 36%, respectively [23]. There was no increased risk of death in patients who received immunotherapy with PD-1 inhibitors. Similarly, another retrospective analysis from the German tumor registry found that 13 patients with metastatic melanoma treated with PD-1 inhibitors were diagnosed with COVID-19, mostly asymptomatic or with mild clinical symptoms, with no exacerbation of irAEs [24]. The safety of PD-1 inhibitors in patients with melanoma and COVID-19 has been confirmed. It has also been advocated to avoid delaying, interrupting, or suspending ICI treatment, including nivolumab and ipilimumab, for melanoma patients due to the COVID-19 pandemic, as the beneficial impact on the long-term survival of nearly 50% of patients outweighs the potential risk of severe SARS-CoV-2 infection [25].

Ahmed [26] reported on an 83-year-old melanoma patient with brain metastases who unfortunately contracted COVID-19 after receiving 7 weeks of ICI treatment (nivolumab and ipilimumab). Although she underwent endotracheal intubation and was transferred to the ICU, the pulmonary consolidation was significantly reduced in the end. A brain magnetic resonance imaging showed tumor regression, and there were no signs of extracranial metastasis in the whole body. It showed that elderly and infirm patients treated with ICI could survive severe COVID-19, and it was also effective against tumors and pneumonia. Another stage IV postoperative melanoma patient presented with mild to moderate cough, sore throat, fever, etc., after infection with SARS-CoV-2, and the fever disappeared within 3 days of nivolumab immunotherapy, had finally recovered completely and remained in complete remission (CR) [27]. Coincidentally, another 75-year-old melanoma patient was reported to have multiple comorbidities and lung metastases who achieved CR at 9 months on nivolumab and was diagnosed with COVID-19 after 27 cycles of ICI with mild symptoms and a mild ground glass opacity (GGO) were left behind [28]. PD-1 inhibitors may cause mild symptoms of COVID-19 in elderly patients with multiple complications.

Similarly, Pala [29] reported a case of a male patient with melanoma accompanied by lung metastasis and no underlying disease who had a long-term response to PD-1 inhibitors and finally recovered after infection with COVID-19. Similarly, a case of a male patient with melanoma accompanied by lung metastasis and no underlying disease showed a long-term response to PD-1 inhibitors, and the patient finally recovered from COVID-19 infection. Computed tomography indicated that pulmonary inflammation subsided without sequelae, suggesting that PD-1 inhibitors may have a potential protective effect against COVID-19. The reason may be that ICI treatment can amplify CD8 effector memory T cells and enhance T-cell activation, thus enhancing T-cell immunity and the anti-SARS-Cov-2 effect [30].

In advanced melanoma, especially in patients with metastatic melanoma resistant to PD-1/PD-L1, ipilimumab combined with PD-1 inhibitors appears to be more effective than ipilimumab alone. Moritz et al. [24] pointed out that among eight patients with advanced melanoma complicated with COVID-19 who received PD-1 inhibitor monotherapy, all but one patient recovered completely from COVID-19. Of the four patients who had complications and received nivolumab combined with ipilimumab, only one developed acute respiratory distress syndrome and the other three did not have serious symptoms of COVID-19, suggesting that the intensity of COVID-19 may vary significantly between individuals. Unfortunately, no difference was observed between ICI monotherapy and combination therapy in this population due to the sample size factors. Combination therapies may be associated with increased toxicity and side effects [31]. For instance, immune-related pneumonia was observed in two patients with metastatic melanoma infected with COVID-19 after combining nivolumab and ipilimumab. However, pneumonia significantly improved after the use of steroids [32].

## 4 Squamous head and neck carcinoma with SARS-CoV-2 infection

Immunotherapy has become a new standard treatment for recurrent and metastatic head and neck cancers [33]. However, in patients with head and neck cancer complicated by COVID-19, there is limited evidence regarding the efficacy and safety of ICI treatment. A case of immune-mediated pneumonia in a 65-year-old patient with head and neck squamous metastatic cancer was reported, who recovered from COVID-19 and received combination therapy, including PD-L1 inhibitors; however, without any clinical manifestations, the pulmonary toxic event was classified as grade 1. When the patient was re-evaluated after the re-initiation of immunotherapy, the tumor achieved PR, and the GGOs appeared unmodified [34]. This study confirmed the safety and efficacy of PD-L1 inhibitors against tumors in patients with head and neck cancer recovering from COVID-19 and that immune-related pneumonia was controllable. However, it has also been suggested that lung damage caused by COVID-19 may increase the risk of immune-related pneumonia, just as other lung injuries and pre-existing pulmonary [35]. This is consistent with the views of Ye et al. [36]. However, for cancer types that are expected to derive substantial clinical benefits from ICI, such as squamous head and neck carcinoma, immunotherapy may be used cautiously for asymptomatic ICI-associated pneumonia based on risk–benefit assessment and the recognized association between irAEs and long-term disease control [21]. Therefore, clinicians should be alert to the coexistence of COVID-19 and immune-related pneumonia during the COVID-19 pandemic.

For patients with platinum-refractory recurrent squamous head and neck carcinoma, ICI treatment based on nivolumab can bring longer survival benefits than standard monotherapy but with a higher incidence of grade 3 and above AEs [37]. To date, no studies have been conducted to compare the advantages and disadvantages of ICI or ICI combination therapy in patients with squamous head and neck carcinoma complicated by COVID-19.

## 5 Hematologic malignancy (HM) with SARS-CoV-2 infection

The first case of COVID-19 in patient with hematologic malignancy was reported from Wuhan, China. Although hospitalized patients with HM had a similar case rate of COVID-19 compared with normal healthcare providers (10 and 7%), the case fatality rate was significantly higher, with 62% for patients with HM compared with 0%, respectively [38]. Studies have shown significant differences in COVID-19 response between HM and solid tumors, for instance, a lower T-cell protective response and a longer RNA persistence beyond 20 days (60 vs. 35%, respectively) [39]. Although ICI has benefited most patients with solid tumors, its efficacy in HMs has been limited, mainly in some subtypes of lymphoma such as classical Hodgkin’s lymphoma and primary mediastinal B-cell lymphoma [40,41]. The application of ICIs in patients with HMs complicated by COVID-19 is not well studied. Hematologic malignancy types are associated with COVID-19 mortality; patients with acute myeloid leukemia and myelodysplastic syndrome are associated with higher COVID-19 mortality rates (44%), whereas Ph-negative MPN and CML are relatively lower (19 and 13%) [42]. A 22-year-old woman with refractory Hodgkin’s lymphoma without any underlying disease was reported to have responded to pembrolizumab during her third cycle and was diagnosed with COVID-19 near the seventh treatment cycle. Due to multiple factors, such as young age and no complications, she was finally discharged from the hospital after positive treatment, which initially proved the safety of ICI in patients with HMs complicated with COVID-19 [43]. However, the therapeutic effects of ICI on COVID-19 have rarely been described.

## 6 Summary

The COVID-19 pandemic has created new challenges for oncology clinicians and cancer patients with COVID-19 receiving immunotherapy. Based on the current clinical problems, this review systematically summarizes the reports of ICI in patients with different cancers complicated by COVID-19, providing more references for ICI application in tumor patients undergoing immunotherapy and COVID-19 patients. According to existing reports, ICI does not appear to increase the risk of infection or exacerbation of COVID-19. Most reports indicate that ICI is safe for patients with tumors complicated with COVID-19. But combined ICI treatment does not benefit patients, but increases the risk of death and incidence of AE. In addition, prolonging the treatment time of ICI or prolonging the administration interval had no significant effect on OS and even could reduce the occurrence of AEs in solid tumor. However, it should be noted that there are significant differences between HMs and solid tumors, including the T-cell response after infection and the duration of RNA, which lead to a worse outcome. This may be related to the complex tumor microenvironment of hematologic tumors and the systemic immune dysfunction of lymphocytes, especially the low response of B cells to vaccines [44]. COVID-19 may increase the risk of immune-related pneumonia in both. Nevertheless, data on the difference between HMs and solid tumors in response to ICI immunotherapy are still lacking. A small number of cases have been reported that ICI treatment was helpful in combating COVID-19 showing with higher levels of SARS-CoV-2-responsive IgG, neutralizing serum activity, and a stronger humoral immune response. Currently, clinical studies on the safety and efficacy of ICI in COVID-19 patients are still being conducted (e.g., NCT04356508, NCT04335305, NCT04413838, NCT04343144), to provide more rigorously controlled study data.
